# *In vitro* and *in vivo* study of the pathogenic role of PPARα in experimental periodontitis

**DOI:** 10.1590/1678-7757-2022-0076

**Published:** 2022-07-08

**Authors:** Ying CHEN, Zheqing JIANG, Ana KEOHANE, HU Yang

**Affiliations:** 1 The Forsyth Institute Department of Immunology and Infectious Diseases Cambridge United States The Forsyth Institute, Department of Immunology and Infectious Diseases, Cambridge, United States.; 2 Boston University Henry M. Goldman School of Dental Medicine Department of General Dentistry Boston United States Boston University Henry M. Goldman School of Dental Medicine, Department of General Dentistry, Boston, United States.

**Keywords:** Periodontits, Peroxisome proliferator-activated receptor alpha, Porphyromonas gingivalis

## Abstract

**Objective:**

The purpose of this study is to investigate the pathogenic role of PPARα in periodontal antigen treated gingival cells *in vitro* and in experimental periodontitis *in vivo* .

**Methodology:**

Gingival fibroblasts, gingival epithelial cells and splenocytes were isolated from C57BL/6J wild type (WT) mice and treated with fixed *P. gingivalis* at for 48 hours. The mRNA levels of PPARs, TNFα, IL-1β and IL-10 were detected by Real-time quantitative PCR. Silk ligatures after being soaked in the *P.gingivalis* suspension were tied around both maxillary second molars of WT mice or PPARα knock-out (KO) mice for two weeks. PPARα agonist fenofibrate and vehicle control were injected into the different side of the palatal gingiva on days 3, 6, and 9. At day 14, bone resorption and gingival mRNA expression levels of PPARs, TNFα, IL-1β and IL-10 were measured by micro-computed tomography and RT-qPCR respectively.

**Results:**

*P. gingivalis* treatment downregulated the expression of PPARα, but not PPARβ or PPARγ, and increased the expression of TNF-α and IL-1β in Gingival fibroblasts, gingival epithelial cells and splenocytes from WT mice. Gingival mRNA levels of PPARα were significantly decreased in experimental periodontitis in WT mice. The bone loss of PPARα KO mice in experimental periodontitis was significantly higher than WT mice and was not reduced by fenofibrate treatment. Gingival TNFα protein expressions were significantly increased by *P. gingivalis* associated ligation and decreased by fenofibrate treatment in WT mice but not in PPARα KO mice.

**Conclusion:**

This study suggests that PPARα plays an essential role in periodontitis.

## Introduction

Periodontitis is a progressive inflammatory disease involved in the teeth surrounding tissues. The inflammation can lead to alveolar osteolysis by increasing the osteoclast activity and inhibiting the osteoblast activity,^1 , 2^ causing tooth loss and jaw bone deterioration in the periodontitis.^3^ Besides local oral cavity pathology, chronic periodontitis is also associated with an increased risk of developing many systemic diseases.^4 , 5^

Inflammation is usually driven by oral bacterial communities interacting with the host immune system and contributes to the inflammation in the process of periodontitis.^6^ Among several main periodontal pathogens, *Porphyromonas gingivalis (P.g)* , a gram-negative oral anaerobe, is one of the most prominent periodontal pathogens.^7^ *P.g* mainly inhabits the subgingival sulcus of human oral cavity and is the main colonizer of dental plaque. The periodontitis is initiated by accumulating plaque, in which the virulence factors are released and induce the immune response. Although the exact mechanism is unknown, it has been hypothesized that the causative role of *P.g* in the periodontitis may be by synthesizing pathogenic factors by its virulence factors.^7^ Lipopolysaccharide (LPS), a key component in the outer membrane of *P.g* , is a causative virulence factor of *P.g.* LPS plays a major pathogenic role that initiate and enhance the inflammation in the periodontitis.^8^ LPS can function as a pathogen-associated molecular pattern to trigger or mediate the inflammatory response by binding to the Toll-like receptor and CD14. Studies have shown that this mechanism contributes to the inflammation and osteoclastogenesis in the periodontitis.^9^

In the periodontitis, the inflammation initiates with the immune response of resident leukocytes to the bacterial biofilm and with the activation of the inflammation cytokines.^10^ TNF-α^11^ and IL-1β^12 , 13^ are the most related pro-inflammatory cytokines and pathways, mediating the pathogenic process of periodontitis. TNF-α is the main connector to higher destructive periodontal disease and is the key pathogen to early inflammatory cytokines. The levels of TNF-α contribute to the onset of destructive periodontal diseases via several mechanisms.^14^ IL-1β triggers many inflammatory reactions such as bone resorption and the production of tissue degrading proteinases. However, the exact mechanism that produces the TNF-α and IL-1β in periodontitis is still unclear.

Peroxisome proliferator-activated receptor alpha (PPARα) is a nuclear hormonal transcription factor and regulates transcription of many genes involved in lipid metabolism, stress response, and inflammation.^15^ PPARs belong to the phylogeny of the steroid receptor superfamily and are called nuclear hormone factors. Its three subtypes include α, β/δ, and γ, and each one mediates many fatty acids (FAs) or FA related actions.^16^ PPARs are activated by binding with lipid-derived ligands, which leads to a transformation change of PPARα, thus forming a complex with retinoid X receptor (RXR), which coordinates gene expression.^15^

PPARα has been less studied compared to PPAR-γ, especially its anti-inflammatory functions. Recent studies have shown that PPARα exerts inflammatory modulation activities by regulating the expression of pro-inflammatory cytokines involved in the inflammatory processes.^17 , 18^ One mechanism is that PPARα interacts with transcription factors or cell signaling to mediate the inflammatory responses. The expression of PPARα appeared in tissues of adipose tissues, liver, kidney, muscle, heart, lung, from abundant to a lesser extent.^18 , 19^ Activation of PPARα inhibits the expression of many pro-inflammatory cytokines and NF-κB signaling in these tissues.^20^ For example, PPARα suppresses Th17 cell differentiation via IL-6 pathway in experimental autoimmune myocarditis.^21^ PPARα inhibits the vascular inflammatory response by interfering with the NF-κB and AP-1 transactivation at a transcriptional level.^22^ Besides the negative regulation of the pro-inflammatory cytokines expression, PPARα can suppress the inflammatory response by upregulating the expression of anti-inflammatory cytokines. For example, studies have shown that the IL-1 receptor antagonist is a direct target gene of PPARα in the liver.^23^

Our study reported that fenofibrate, a PPARα activator, decreases inflammatory cytokines by inhibiting the TLR/NF-κB, signaling a pathway in uveitis, an ocular presentation of the systemic inflammatory disease and that PPARα is essential for this anti-inflammatory effect.^24^ Activation of PPARα by fenofibrate reduced TNF-α production and NF-κB nuclear transaction. There are several studies of PPARs in the periodontitis, for example, Taskan’s study reported that the PPARγ level increased in patients with periodontitis,^25^ whereas Briguglio’s study of WY-14643, a potent PPARα agonist, in an experimental rat model showed that the inflammatory process associated with experimental periodontitis improved.^26^ We assessed the pathogenic role of PPARα and the effects of PPARαactivation/defect on inflammation and bone loss in periodontitis.

## Methodology

### Animal

Wild-type (WT) C57BL/6 mice and PPARα KO mice (Jackson Laboratory) of 8-10 weeks old were used for experiments with an equal amount of male and female mice. A total of 12 mice (two groups, six per group) were used for *in vitro* study and a total 36 mice (six groups, six per group) were used for *in vivo* study. The sample size for *in vitro* study was determined by a power analysis performed with JMP Pro 13 statistical software based on preliminary data on alveolar bone loss. If α is set to 0.05 and β to 0.2 (which allows for 0.8 power), six samples are needed per treatment variable. All mice were randomly distributed into all groups across multiple cages and litters, with the mouse cages on the racks. This experimental periodontitis model showed no losses or adverse effects. All the animal experiments have been approved by the Institutional Animal Care and Use Committee.

### Cell preparation and culture

Mouse splenocytes were isolated and cultured from spleen from WT mice and PPARα KO mice.^27^ Mouse gingival fibroblasts obtained from WT mice were cultured following a protocol previously described.^28^ The gingival tissue was cut, minced and digested with collagenase I (2 mg/mL, Worthington Biochemical, Lakewood, NJ). After filtered by a nylon mesh filter and centrifuged for 10 min at 1,500 rpm, raw cells pellets were re-suspended and plated at a density of 10^5^ cell/cm^2^. Cells were passaged at a 70% confluence. Fibroblasts between passages 3–6 were used for experiments. Mouse gingival epithelial cells were isolated.^29^ The palatal gingival tissue was harvested and minced by scalpel and then digested in solutions containing Dispase II (2mg/ml, Sigma) and collagenase (4mg/ml, Sigma) for 1.5 hours at 37^ο^C. After centrifugation and rinse, the cells were cultured in Keratinocyte Serum-Free Medium (Gibco) for seven days and ready to use. Primary gingival fibroblasts, gingival epithelial cells and splenocytes were treated with *P. gingivalis* at a dosage of 5x10^5^ per 1×10^6^ cells for 48 hours in the absence or presence of LPS (L6529; Sigma-Aldrich, St. Louis, MO) (1µg/ml), LPS(1µg/ml)+Fenofibrate (50μM), LPS(1µg/ml)+WY14643 (Sigma, St. Louis, MO)(PPARα agonist, 100μM), LPS(1µg/ml)+WY14643(100μM)+GW6471 (Sigma, St. Louis, MO)(PPARα antagonist, 10μM). Mouse splenocyte of PPARα KO mice were infected with Ad-GFP (control vector) or Ad-PPARα at multiplicity of infection (MOI) of 20 for 24 hours, then treated with LPS (1µg/ml), LPS(1µg/ml)+Fenofibrate (50μM) for 48 hours.

### Animal model and local administration

The *P.gingivalis* -associated ligature-induced experimental periodontitis was induced with silk (7-0, Fisher Scientific) after being soaked in the *P.gingivalis* (Strain ATCC 33277) suspension for 30 minutes, following a ligation around maxillary second molar for two weeks in WT mice or PPARα KO mice.^30^ Group 1(n=6 animals/group) had no ligation on both sides. Group 2 (n=12 animals/group) had both maxillary second molars ligatured: the left side injected with fenofibrate (50 µM, 2 µl) and the right side injected with vehicle (PBS, 2 µl) on day 3, 6, 9 during the ligation. All the mice were sacrificed on day 14.

### Real-time PCR

Total RNA was extracted from cultured gingival fibroblasts, gingival epithelial cells and splenocytes or palatal gingival tissues using the PureLink^®^ RNA Mini Kit (Ambion). cDNA was synthesized using the SuperScript II Reverse Transcriptase kit (Invitrogen) and amplified by quantitative real-time PCR.^30^ The following primers were used: TNF-α forward 5’-CACAGAAAGCATGATCCGCGACGT-3’; TNF-α reverse 5’-CGGCAGAGAGGAGGTTGACTTTCT-3’; IL-1βforward5’-CCAGCTTCAAATCTCACAGCAG-3’; IL-1βreverse5’-CTTCTTTGGGTATTGCTTGGGATC-3’; IL-10 forward5’-CTTCTTTGGGTATTGCTTGGGATC-3’;IL-10 reverse5’-CAGCAGACTCAATACACACT-3’;GAPDH: F: AGCAGTCCCGTACACTGGCAAAC, R: TCTGTGGTGATGTAAATGTCCTCT.

### Bone morphometric analysis

The bone loss of experimental periodontitis mice was measured by a high-resolution scanner (mCT-40, Scanco Medical) and analyzed via Seg3D software.^27^ The same volume of interest (VOI) was chosen for each sample around the second maxillary molar. A cylinder with a diameter of 1.0 mm and a height of 1.0 mm is defined as VOI of the top surface of the natural tooth. A 3D morphometric analysis was conducted to determine the architecture of the bone based on total VOI volume (TV) and total bone volume (BV). The empty space volumes (ESV) surrounding tooth or implants were estimated by TV minus BV. The micro-CT images of implant and natural tooth were converted and collected by the Amira software (FEI Visualization Sciences Group).

### Western blot analysis

A procedure was followed as previously described.^31^ The same amount (50 µg) of gingival proteins or total cell lysates were used to analyze TNFα and the images were semiquantified by densitometry and normalized by β-actin levels via Image J software. Primary antibodies were rabbit anti-TNFα antibody (Abcam, ab6671,1:500), rabbit anti-PPAR alpha antibody (Abcam, ab126285, 1:500), and rabbit anti-β-actin (Abcam, ab8227, 1:2000).

### Statistical analysis

All the quantitative data were expressed as means ± SD. Unpaired Student’s *t* test was performed to compare both groups of datasets in statistical analysis. For multiple groups, differences were analyzed using the one-way analysis of variance (ANOVA) test followed by SNK- *q* multiple comparisons using GraphPad 6.0 software (La Jolla, CA). Statistical significance was set at p<0.05.

## Results

### *P. gingivalis* downregulated the expression of PPARα, but not PPARβ or PPARγ *in vitro* .

**Figure1:**
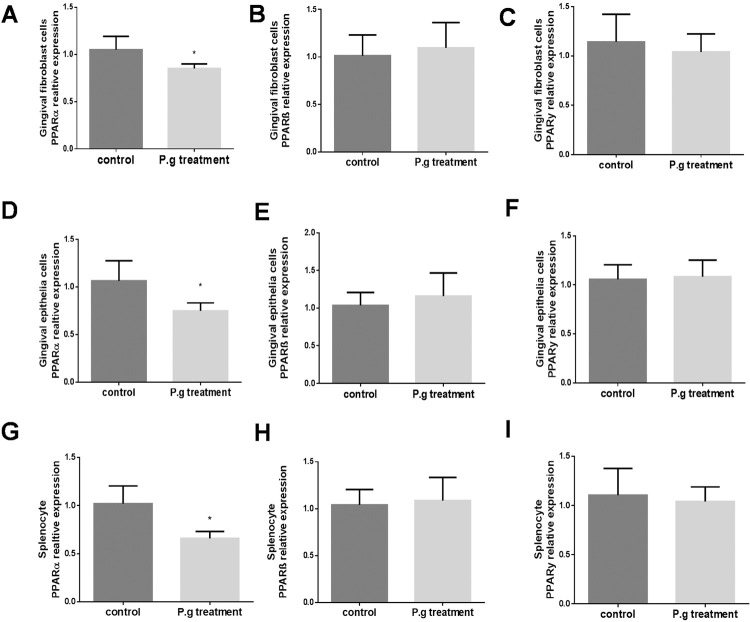
Downregulation of PPARα mRNA expression in *P. gingivalis* treated gingival fibroblasts, gingival epithelial cells, and splenocytes of WT mice. Primary gingival fibroblasts, gingival epithelial cells, and splenocytes were separated from WT mice and treated with fixed *P. gingivalis* at dosages of 5×105 per 1×106 cells for 48 hours. Real-time PCR was performed to measure mRNA levels of PPARα, PPARβ, and PPARγ, respectively, in gingival fibroblasts (A, B, C), gingival epithelial cells (D, E, F) and splenocytes (G, H, I) (mean±SD, all groups n=4, *p<0.05)

The first experiment is to assess if the PPARs are expressed in the different types of cells related to periodontitis, including gingival fibroblasts, gingival epithelial cells and immune cells (mimicked by splenocytes). Primary cultured gingival fibroblasts, gingival epithelial cells, and splenocytes were isolated from mice and cultured. The expressions of PPARs were quantified by quantitative real-time PCR. Figure 1 shows that all types of PPARs, including PPARα, PPARβ, and PPARγ, were expressed in these three types of cells. Then we tested if the expression of PPARs changed in periodontal conditions and if the *P. gingivalis* contributes to these changes. Primary cultured gingival fibroblasts, gingival epithelial cells, and splenocytes were exposed to *P. gingivalis* , a condition that mimics periodontitis. We quantified the transcriptional levels of PPARs of each group and compared to their controls. Figure 1A , 1D , and 1G show that exposure to *P. gingivalis* significantly decreased PPARα expression in all three cells. The reduction of PPARα in splenocytes was greater than in gingival cells. However, unlike the decreases of PPARα, the expression of PPARβ ( Figure 1 B , 1E , and 1H ) and PPAR gamma ( Figure 1C , 1F , and 1I ) was not significantly affected in all three types of cells. These results suggested that the PPARα, not PPARβ and PPAR gamma, is associated with the infection of periodontal pathogen.

### *P. gingivalis* induced pro-inflammatory cytokines *in vitro* .

**Figure2:**
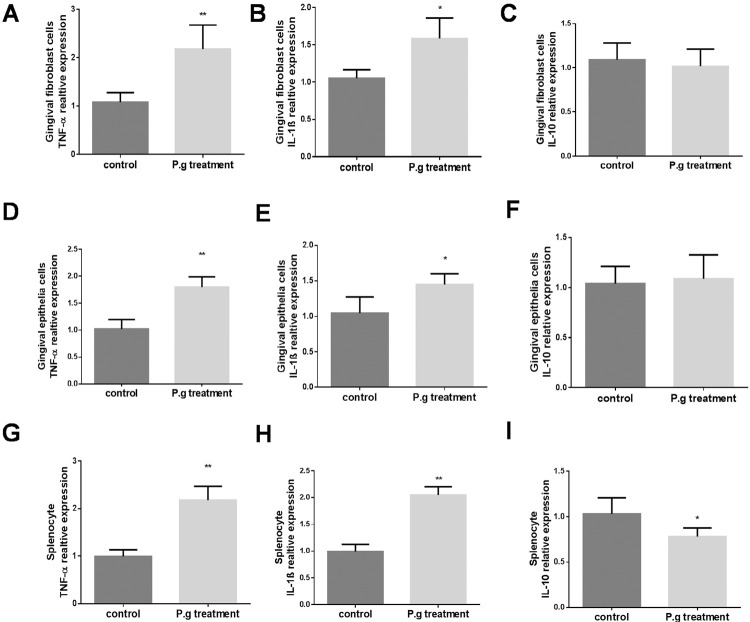
Effects of *P. gingivalis* treatment on mRNA expression of inflammatory cytokines in gingival fibroblasts, gingival epithelial cells, and splenocytes. Primary gingival fibroblasts, gingival epithelial cells, and splenocytes were separated from WT mice and treated with fixed *P. gingivalis* at dosages of 5×105 per 1×106 cells for 48 hours. Real-time PCR was performed to measure mRNA levels of pro-inflammatory cytokine TNFα, IL-1β and anti-inflammatory cytokine IL-10, respectively, in gingival fibroblasts (A, B, C), gingival epithelial cells (D, E, F) and splenocytes (G, H, I) (mean±SD, all groups n=4, *p<0.05, ** p<0.01)

Since *P. gingivalis* infection is associated with a progressive increase of pro-inflammatory cytokines in periodontal diseases, we examined the effect of *P. gingivalis* treatment on the expression of pro-inflammatory cytokines TNF-α and IL-1β, the two main cytokines present in diseased periodontal tissues. The data showed that exposure to *P. gingivalis* significantly increased the expression of TNF-α ( Figure 2A , 191% vs. control group) and IL-1β ( Fig2B , 158% vs. control group) in the gingival fibroblasts, which is similar in gingival epithelial cells ( Figure 2D , 2E , 187% and 148% vs. TNF-α and IL-1β, respectively) and splenocytes ( Figure 2G , 2H , 208% and 201% vs. TNF-α and IL-1β, respectively). Unlike the increases of TNF-α and IL-1β, the anti-inflammatory cytokine IL-10 was not significantly changed in gingival fibroblasts and in gingival epithelial cells ( Figure 2C and 2F ). Unlike the increase in IL-1β and TNF-α and no change of IL-10 in the gingival cells, IL-10 significantly decreased ( Figure 2I 78% vs. control group) in splenocytes, which indicates a possible different function of IL-10 in the gingival cells and splenocytes.

Gingival PPARα decreased and pro-inflammatory cytokines increased in *P.gingivalis* -associated ligature-induced experimental periodontitis.

**Figure 3:**
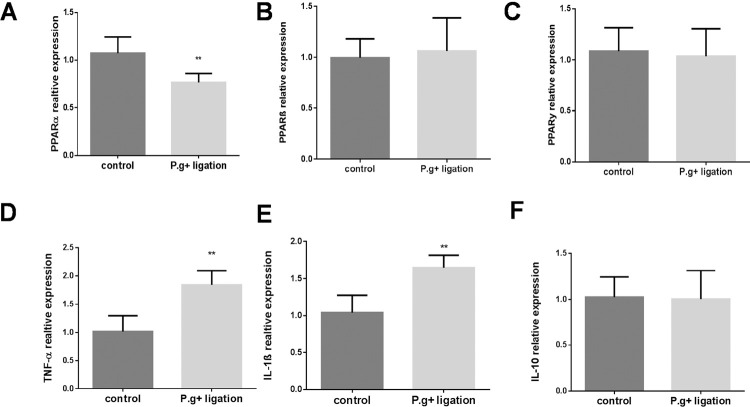
Gingival mRNA expression of PPARs and inflammatory cytokines in *P. gingivalis* -associated ligature-induced experimental periodontitis. After being soaked in the P.gingivalis suspension, silk ligatures were tied around maxillary second molars of left sides in WT mice on day 0 and last for 14 days. The gingival tissue mRNA levels of PPARα (A), PPARβ (B), and PPARγ (C), TNF-α (D), IL-1β (E), IL-10 (F) were measured and analyzed. (mean±SD, all groups n=5, *p<0.05, **p<0.01)

Then we examined the expression of gingival PPARα and pro-inflammatory cytokines in periodontitis WT mice. Figure 3 shows that two weeks ligation of *P. gingivalis* -soaked silk significantly decreased PPARα expression ( Figure 3A , 71% vs. control group) but not PPARβ ( Figure 3B ) and PPARγ ( Figure 3C ), it increased the expressions of TNF-α ( Figure 3G , 193% vs. control group) and IL-1β ( Figure 3H , 179% vs control group) in the gingival tissues, and the expression of gingival IL-10 did not significantly changed ( Figure 3F ). These results agree with the finding in the gingival fibroblasts and epithelial cells ( Figure 1 and Figure 2 ).

### PPARα protects the bone in experimental periodontitis.

**Figure 4:**
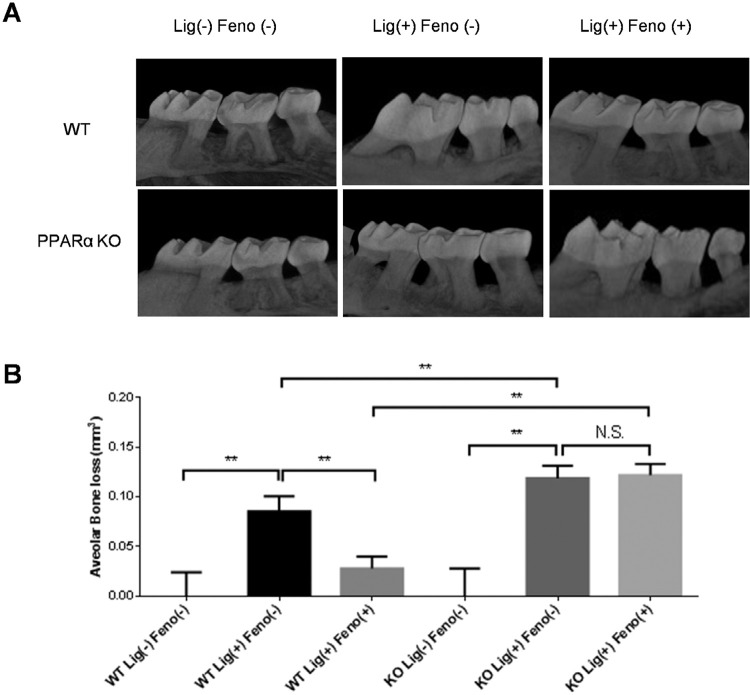
Bone loss comparisons between WT and PPARα mice with/without PPARα treatment in *P. gingivalis* -associated ligature-induced experimental periodontitis. After being soaked in the *P. gingivalis* suspension, silk ligatures were tied around maxillary second molars of both sides in C57/BL6 mice on day 0 and fenofibrate (50 µM, 2 µl) or vehicle (PBS, 2 µl) was injected on days 3, 6 and 9 in WT mice and PPARα KO mice. Maxilla were collected on day 14 and measured by 3D micro-CT (A) and analyzed as bone resorption volume/mm3 (B) (mean±SD, n=5, **p<0.01, N.S., no significance)

Since periodontitis is characterized by inflammation and bone loss, inflammation is a causative pathogen in bone resorption and is related to bone loss. We examined the PPARα’s bone protection effect in the periodontitis mice. Two weeks of *P. gingivalis* -associated ligation induced a significant bone resorption in the WT mice, and bone loss significantly increased in PPARα KO mice compared to WT mice ( Figure 4A , 4B ). Moreover, PPARα agonist fenofibrate treatment significantly reduced the bone loss in WT mice but not in PPARα KO mice ( Figure 4A , 4B ), indicating that PPARα is essential to protect the bone in an experimental periodontitis model.

### PPARα is essential to regulate the production of TNF-α induced by *P. gingivalis in vivo* and *in vitro* .

**Figure 5:**
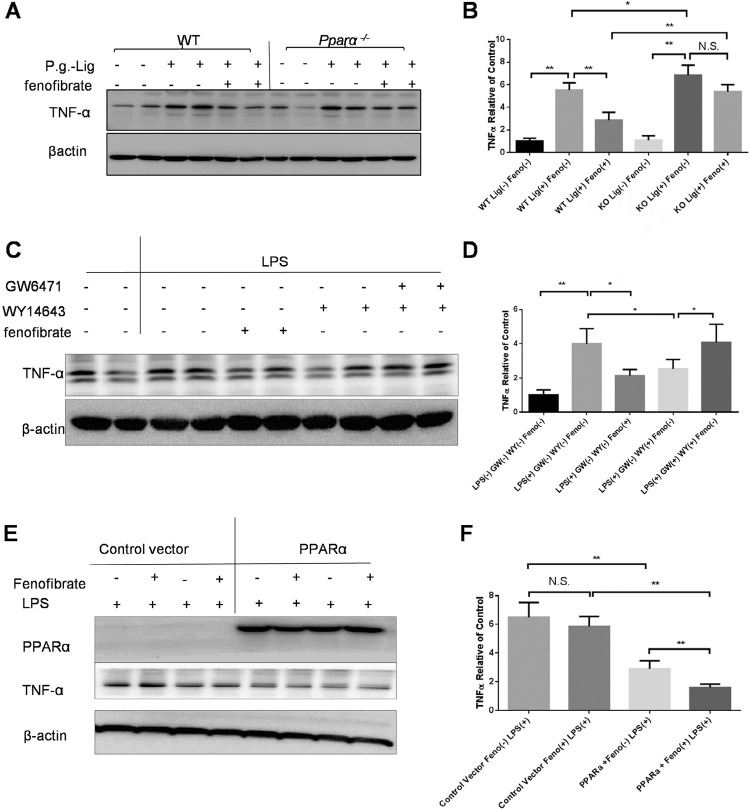
PPARα is essential to regulate the pro-inflammatory marker TNFα in ligature-induced experimental periodontitis. The same amount (50 µg) of gingival proteins from sites without ligation, ligation with vehicle treatment, ligation with fenofibrate treatment of WT and PPARα mice were used for Western blot analysis of TNFα (A), semiquantified by densitometry and normalized by β-actin levels (B) (mean±SD, n=4, *p<0.05, **p<0.01, N.S., no significance). Mouse splenocytes were separated from WT mice and treated with LPS (1µg/ml), LPS(1µg/ml)+fenofibrate (50μM), LPS(1µg/ml)+WY14643(100μM), LPS(1µg/ml)+WY14643(100μM)+GW6471(10μM) for 48 hours. Total cell lysates were used for Western blot analysis of TNFα (C), semiquantified by densitometry and normalized by β-actin levels (D) (mean±SD, n=4, *p<0.05, **p<0.01). Mouse splenocytes were separated from PPARα mice and infected with adenovirus with control vector or PPARα vector for 24 hours and then treated with LPS (1µg/ml), LPS(1µg/ml)+fenofibrate (50μM) for 48 hours. Total cell lysates were used for Western blot analysis of TNFα (E), semiquantified by densitometry and normalized by β-actin levels (F) (mean±SD, n=4, **p<0.01, N.S., no significance)

We examined the effect of PPARα on the expression of pro-inflammatory cytokines *in vivo* and *in vitro* to determine if increases of the pro-inflammatory cytokines are associated with the PPARα reduction. Firstly, we evaluated the anti-inflammatory effects of fenofibrate in the experimental periodontitis mice. Figure 5A and 5B show that *P. gingivalis* ligature increased TNF-α protein expression in the gingival tissues of both WT mice and PPARα KO mice. These increases were largely reduced by fenofibrate in the WT mice, but not in PPARα KO mice ( Figure 5A , 5B ). This result agrees with the *in vitro* findings ( Figure 3D ), which suggests that PPARα partly reverses TNF-α production while KO mice produce higher levels of TNF-α than WT.

Then we examined if PPARα has a similar TNFα regulating effect on immune cells under periodontitis conditions. The splenocytes of WT mice were exposed to LPS with/without the presence of fenofibrate. Figure 5C and 5D show that exposure to LPS increased TNFα, which was significantly reduced by fenofibrate. We replaced fenofibrate by WY14643 in a parallel experiment, a synthesized PPARα agonist, to see if activation of PPARα by other agonist can reduce the expression of TNFα. The elevation of the TNFα induced by the LPS was significantly decreased by WY14643, a similar effect as the fenofibrate ( Figure 5C , 5D ). We added GW6741, a PPARα antagonist, to the treatment of the cell to antagonize the PPARα activation by WY14643 to determine if this reduction was by PPARα. The level of TNFα was completely reversed to the level comparable to the LPS control, suggesting that the effect of fenofibrate on the TNF-α is likely via a PPARα-dependent mechanism in WT splenocyte ( Figure 5C , 5D ).

Furthermore, splenocytes from PPARα KO mice was infected by an adenovirus with GFP (control vector) or PPARα for 24 hours and then exposed to LPS with or without the presence of fenofibrate treatment. The data showed that LPS induced TNFα, but fenofibrate treatment did not suppress this induction when PPARα was knock out ( Figure 5E , 5F ). However, the induction levels of TNFα by LPS was significantly lower than PPARα knockout conditions when PPARα was re-expressed in KO cells and fenofibrate treatment reduced the induction of TNFα ( Figure 5E , 5F ), similar to WT splenocytes ( Figure 5C , 5D ). This result suggested that the reduction of TNF-α in periodontitis may depend on PPARα.

## Discussion

Our study assessed the pathogenic role of PPARα in periodontitis. The data showed that PPARα was decreased and pro-inflammatory cytokines were increased by *P. gingivalis* in the gingival cells and splenocytes. *In vitro* and *in vivo* studies showed that fenofibrate reduced IL-1 and TNFα by PPARα-dependent mechanisms. As IL-1 and TNFα were elevated by ligature-induced in both WT and PPARα KO mice, treatment with fenofibrate reduced the production of pro-inflammatory cytokines in WT mice but not in PPARα KO mice. Lack of PPARα caused higher alveolar bone loss and fenofibrate treatment prevented alveolar bone loss in a PPARα-dependent mechanism.

Our most essential finding is that PPARα plays a crucial role in periodontitis. We emphasize that PPARα, not PPARß or PPRAα, is affected by the *P. gingivalis* ligation. We evidence that the transcriptional level of PPARα decreased in the presence of *P. gingivalis* in the gingival fibroblasts, gingival epithelial cells, and splenocytes, and decreased in the gingival tissue from experimental periodontitis mice as well. Despite many studies reported that PPARα expression was lower in the healthy group and higher in periodontitis and peri-implantitis patients,^25^ we have not observed significant changes of PPARβ or PPRAα at the transcriptional level in the *P. gingivalis* + ligation conditions. Whether this elevation of PPRAα is related to another non-transcriptional mechanism or is a compensation or feedback at the late stage remain unclear.

Cytokines such as IL-1β and TNFα have been reported to play destructive roles on soft tissue and bone resorption.^32^ Our study showed that the transcription of IL-1βand TNF-α were upregulated in gingival and spleen cell and tissues in the presence of *P. gingivalis* with ligation, which agrees with published data associating periodontitis with increases of IL-1β and TNF-α,^32^ essential to mediate the pathogenesis of tissue destruction and bone loss. The levels of IL-10 did not significantly change in gingival cells. In the splenocytes, the IL-10 levels were decreased by exposure to the periodontal condition, which suggests a likely protective function of IL-10 in the immune cells.

Our study shows the association between PPARα and the periodontitis cytokines. PPARα is an essential regulator of TNFα in periodontal tissues, which is supported by 1) activation of PPARα decreased LPS-induced elevation of TNFα in the splenocytes of WT mice; 2) over-expression of PPARα reduced the LPS-induced TNFα elevation of the splenocytes of PPARα KO mice; 3) the PPARα’s anti-TNFα effect was antagonized by the PPARα antagonist; 4) PPARα’s anti-TNFα effect was futile in PPARα KO mice. Fenofibrate, one of the commonly used lipid-lowering drugs, is a PPARα activator. Studies have shown that activation of PPARα with fenofibrate improves inflammation in many diseases. Our previous study showed that fenofibrate reduced pro-inflammatory cytokines in LPS-induced acute uveitis by downregulating TLR4 in the RPE and iris.^24^ LPS from *P. gingivalis* is is a key factor to develop periodontitis and LPS is the agonist of TLR4, however, it is unclear if fenofibrate reduces the production of pro-inflammatory cytokine in the periodontitis related to downregulating the TLR4, which requires further studies.

IL-1β and TNFα regulate osteoclast formation and activity, and blocked IL-1β and TNFα with antagonists inhibit bone loss in experimental periodontitis.^33^ Our study shows that fenofibrate improved the alveolar bone resorption and bone loss in the *P. gingivalis* -associated ligature-induced periodontitis mice. Similar to PPARα-dependent anti-inflammatory effect, this bone protective effect was not shown in PPARα KO mice. However, it is unclear whether this bone protective effect of fenofibrate was due to improvement of the inflammation or to a PPARα osteo-related effect. All the PPARs are expressed in bone cells. However, the effects of PPARs on the bone were different. Both PPARβ and PPARγ inhibit bone formation and stimulate bone resorption, whereas PPARα showed a skeletal protective effect. For example, Syversen’s study of female rats showed that feeding with fenofibrate had higher femoral BMD and smaller medullary area and normal trabecular bone volume, while treatment with pioglitazone (PPAR gamma agonist) caused a lower whole-body BMD, BMC, and trabecular bone volume.^34^ The mechanism by which PPAR β and gamma mediate the bone loss and bone impairment include affecting the OPG/RANKL/RANK system,^35 - 37^ suppressing osteoblast differentiation from mesenchymal stem cells by favoring adipogenesis,^38^ inducing osteocyte apoptosis,^37^ and enhancing osteoclastic gene expression.^39^ PPARα controls a wide range of gene transcription in regulating biological processes. PPARα has also coordinated with a vitamin D receptor a cross-regulation of bone target gene transcription. It remains unclear if these PPARα target genes antagonize the bone impairing mechanism of PPARγ and PPARβ mediation or if PPARα activation has a different bone regulating mechanism. Future study is needed to address that.

Our study has many limitations that must be considered for future research. First, all these experiments were performed in mouse cells and mouse experimental model. Whether PPARα has a similar pathogenic role in human patients with periodontitis is unclear and must be addressed. Secondly, although TNFα are the main pro-inflammatory cytokines in experimental periodontitis model, other cytokines such as IL-6 and INF-γ must be studied with PPARα activation in the future. Moreover, mouse splenocytes contain 21%-25% T cells, 44%-58% B cells, 3.5%-5% monocyte, and about 1% of macrophages and other immune cells. It may not reflect the real composition of infiltrated immune cells in gingival tissue under periodontitis condition. Thus, using splenocytes to study oral infiltrated immune cells has its limitation. Furthermore, five main mechanisms were already known for fibrates that extrapolate peroxisome proliferation. However, the anti-inflammation and anti-angiogenesis function of fenofibrate was recently studied in diabetic retinopathy. Their mechanisms need further study. Finally, we only used one optimized dose of fenofibrate (50 µM). Although our previous publication showed that the 50 μM dose had the most effective anti-inflammation effect reported in the *in vitro* study,^40^ multiple doses will give more information to extend the study.

Our study shows that PPARα is an essential transcription factor to regulate inflammation and bone loss in the *P. gingivalis* -associated ligature-induced experimental periodontitis. Activation of PPARα downregulated the expression and production of the pro-inflammatory cytokines, and inhibited the alveolar bone loss, which represents a new therapeutic target to treat periodontitis.
